# Chemical Characterization and Antibiotic-Enhancing Activity of the Essential Oils of Propolis of *Melipona quadrifasciata quadrifasciata*

**DOI:** 10.3390/plants14040587

**Published:** 2025-02-14

**Authors:** Emílio Sousa Albuquerque, Cicera Laura Roque Paulo, João Arthur de Oliveira Borges, Sheila Alves Gonçalves, Gabriel Gonçalves Alencar, Judith Ferreira do Carmo, Angelica Isabely de Morais Almeida, Maura Lins dos Santos, José Weverton Almeida-Bezerra, Luiz Everson da Silva, Cícera Datiane de Morais Oliveira-Tintino, Henrique Douglas Melo Coutinho

**Affiliations:** 1 Laboratory of Microbiology and Molecular Biology (LMBM), Regional University of Cariri (URCA), Crato 63105-000, Brazil; emilioalbuq@gmail.com (E.S.A.); datianemorais@hotmail.com (C.D.d.M.O.-T.); 2 Master’s Program in Health Education, Doctor Leão Sampaio University Center, Juazeiro do Norte 63041-140, Brazil; 3 Center for Biological and Health Sciences (CCBS), Nursing Department, Regional University of Cariri (URCA), Crato 63105-000, Brazil; 4 Master’s Program in Sustainable Territorial Development, Federal University of Paraná (UFPR), Curitiba 80060-000, Brazil; 5 Postgraduate Program in Sustainable Territorial Development, Coastal Sector, Federal University of Paraná (UFPR), Litoral Campus, Matinhos 83260-000, Brazil

**Keywords:** biological activity, chemical composition, *Melipona quadrifasciata quadrifasciata*, essential oil, propolis

## Abstract

This study investigated the chemical composition and potential antibacterial activity of the essential oils from the propolis of *Melipona quadrifasciata quadrifasciata* (MQP) in samples collected from the cities of Paranaguá and Guaratuba, Paraná, Brazil, during summer and winter. The chemical composition of the oils was analyzed by GC-MS, and their minimum inhibitory concentration (MIC) was determined against standard ATCC strains and some clinical isolates (*Escherichia coli* 06 and *Staphylococcus aureus* 10). The essential oils’ MICs were determined using microdilution in 96-well plates and showed no significant antibacterial activity (MIC ≥ 1024 μg/mL) when used alone. However, the essential oils enhanced the action of norfloxacin, gentamicin, and ampicillin, especially against S. aureus 10. The chemical analysis identified 68 compounds, with β-bisabolene and β-caryophyllene as the predominant constituents. Subsequently, the antibiotic-enhancing activity against the clinical isolates was evaluated. The composition of the MQP oils varied according to seasonality and location, influenced by the microclimates of the cities. The results highlight the importance of MQP oils in enhancing antibiotic activity, particularly against Gram-positive bacteria, despite the lack of significant standalone antibacterial activity.

## 1. Introduction

Bacterial infections have been the third leading cause of death worldwide over the last five years, following only ischemic heart diseases and health complications related to COVID-19. These bacteria are associated with approximately 33 types of infections, causing around 7.7 million deaths. The five main pathogenic agents responsible for these infections are *Staphylococcus aureus*, *Escherichia coli*, *Streptococcus pneumoniae*, *Klebsiella pneumoniae*, and *Pseudomonas aeruginosa*. These pathogens account for 54.9% of deaths, with mortality rates varying by region, based on data from the Global Burden of Disease Study [1,2].

Researchers have emphasized that, by 2050, multidrug-resistant bacteria could cause severe infections with global economic impacts, potentially compromising essential medical treatments, such as surgeries and therapies for chronic diseases, leading to a loss of USD 100 trillion in the global economy [3].

The indiscriminate use of antimicrobials has led to the development of drug-resistant bacteria, a situation exacerbated during the COVID-19 pandemic [4]. In this context, Murray et al. (2022) [5] reported that, in 2019, approximately 4.95 million deaths were related to infections caused by drug-resistant bacteria, of which 1.27 million were directly attributed to drug resistance.

Amid this concerning scenario, the search for new drugs to combat resistant bacteria has intensified. However, challenges remain in finding new drugs with effective action spectra against these bacteria. The 2020 WHO report highlighted that none of the 43 antibiotics under clinical development adequately addresses the problem of resistant bacteria [6].

In this context, the use of natural products as promising precursors in the search for new antimicrobial agents should be emphasized. Among these products, essential oils stand out as complex mixtures of volatile and lipophilic substances, scientifically noted for their activity against certain bacterial strains of *S. aureus* [7,8,9].

The antibacterial activity of essential oils is attributed to the presence of terpenoids, their main constituents. Their use as antibacterial agents is due to their favorable pharmacokinetic properties and suitable molecular weight [10].

Essential oils exhibit varied mechanisms of action, allowing them to interact with different cellular structures [11,12]. One natural product with a rich essential oil composition is propolis, which is primarily derived from plant resins collected by bees and is produced by mixing wax with small amounts of saliva, forming the final substance. It is a resinous substance collected by bees from buds, barks, and other parts of plants [13].

The composition of propolis is variable according to the literature, and there is no consensus regarding this composition. Some studies cite traditional figures of 50–60% resins and balsams, 30–40% waxes, 5–10% oils, and 5% pollen grains, but these data do not accurately reflect its true composition. When quantification studies are conducted, it is observed that resin content routinely exceeds 70%, while wax content in propolis generally remains below 10%. Additionally, volatile oils are found in low concentrations, often not exceeding 1%. As for pollen grains, there are currently no established methods to quantify their mass in propolis samples [14,15].

The extraction of essential oils from propolis has shown significant potential in recent research, particularly for its antimicrobial and antioxidant activities. It is important to note that the chemical compounds in propolis vary depending on the regions where it is collected and the plant species near bee hives [16,17].

As an example of propolis-producing bees, the stingless bee *Melipona quadrifasciata quadrifasciata* (MQP) (Lepeletier, 1836) [18], commonly known as “mandaçaia”, can be found in much of Brazil’s coastal regions. Previous studies on the chemical composition of MQP propolis have identified compounds with biological activities such as antimicrobial, antioxidant, and cytotoxic effects [19,20].

It is essential to explore the variations in the chemical composition of propolis produced by *M. quadrifasciata quadrifasciata*, which may occur depending on the region and the season of its production and collection. Therefore, this study aims to identify the different compounds in MQP essential oils according to their production in distinct locations and seasons (winter and summer) to explore their potential as antibacterial agents and antibiotic activity enhancers.

## 2. Materials and Methods

### 2.1. Essential Oils of Propolis from Melipona quadrifasciata quadrifasciata

**Figure 1:**
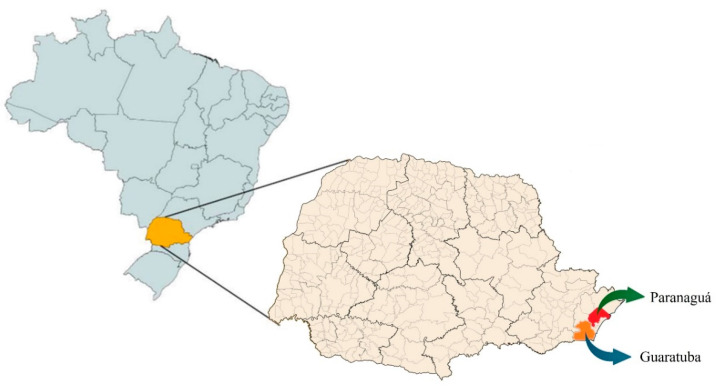
Geographic location of Paranaguá and Guaratuba, Paraná, Brazil. Figure prepared by the authors (2025).

The essential oils were extracted from propolis produced by the species *M*. *quadrifasciata quadrifasciata* (MQP), commonly known as “mandaçaia”, collected from two locations, Paranaguá and Guaratuba (Figure 1), both in the state of Paraná, southern Brazil. The collection sites were located at 25°31′12″ S, 48°30′32″ W and 25°52′58″ S, 48°34′30″ W, respectively. The samples were collected during winter and summer, yielding four samples: WP—Winter Paranaguá; SP—Summer Paranaguá; WG—Winter Guaratuba; SG—Summer Guaratuba (Figure 1) (Table 1).

#### 2.1.1. Extraction of Propolis Essential Oil

The method for extracting MQP essential oils began by crushing the *M. quadrifasciata quadrifasciata* propolis to increase its surface contact with the solvent. The process then dissolved approximately 100 g of each sample in 50 mL of distilled water, allowing it to rest for 20 min. Afterward, the solution was diluted with 350 mL of distilled water. Fresh material from each collection underwent volatile fraction extraction using hydrodistillation in a Clevenger-type apparatus. The process lasted four hours, during which the volatile material was collected with a pipette and stored in microtubes in a freezer [21].

#### 2.1.2. Chemical Composition Analysis

The volatile fractions of the *M. quadrifasciata quadrifasciata* propolis were analyzed chemically using gas chromatography–mass spectrometry (GC-MS). The samples were prepared according to a protocol for chromatographic analysis using a Shimadzu GCMS-TQ8040 (Shimadzu Analytical Pvt. Ltd., Mumbai, India) apparatus and an SH-Rtx-5 ms column (30 m, 0.25 mm, 0.25 μm, temperature range of 60 °C to 300 °C, with an increase of 3 °C per minute), split 90, coupled with a mass spectrometer to determine the chemical composition.

The electron ionization mode (70 eV) was used for mass detection at a scanning rate of 3.15 min^−1^, with a mass range of 40–450 u. The transfer line was maintained at 260 °C, the ion source at 230 °C, and the quadrupole analyzer at 150 °C. The samples were injected into an Agilent 7890A (Agilent Technologies, Shanghai, China) chromatograph equipped with a flame ionization detector (FID) operated at 280 °C for quantification. The same column and analytical conditions described above were used, except for the carrier gas, which was hydrogen at a flow rate of 1.5 mL min^−1^.

The percentage composition was obtained by electronically integrating the FID signal, dividing the area of each component by the total area (area %). The constituents were identified by comparing their mass spectra with those in the Adams library (2007) [22] and their linear retention indices calculated from the injection of a homologous series of hydrocarbons (C7–C26). These were compared with data from the literature, and the peaks were analyzed using “GCMS Postrun Analysis” software (version 4.41).

### 2.2. Antibacterial Activity

#### 2.2.1. Bacterial Strains

The antibacterial activity tests used standard bacterial strains and clinical isolates. For determining the minimum inhibitory concentration (MIC), the standard strains *Escherichia coli* ATCC 25,922 and *Staphylococcus aureus* ATCC 25,923 were utilized. Multidrug-resistant strains of *E. coli* 06 and *S. aureus* 10 were employed for evaluating the antibiotic-enhancing effects. These resistant strains were derived from clinical isolates and displayed multidrug resistance profiles (Table 2).

#### 2.2.2. Bacterial Inoculum Preparation

The bacteria were cultured in solid Heart Infusion Agar (HIA) medium prepared according to the manufacturer’s instructions. After incubation, bacterial colonies were transferred to sterile test tubes containing 4 mL of isotonic saline solution (0.9% NaCl). The tubes were vortexed to ensure uniform bacterial distribution, and the turbidity of the suspension was adjusted to match the 0.5 McFarland scale (1.5 × 10^8^ CFU/mL), ensuring a standardized bacterial concentration [24].

#### 2.2.3. Substances Used

Fractions of 10 mg of MQP oils and the antibiotics norfloxacin (fluoroquinolone), gentamicin (aminoglycoside), and ampicillin (penicillin) were diluted in 0.5 mL of DMSO (dimethyl sulfoxide) and 9265 μL of sterile distilled water to achieve a concentration of 1024 μg/mL. The antibiotics were sourced from Sigma-Aldrich Co. (St. Louis, MO, USA).

#### 2.2.4. Minimum Inhibitory Concentration (MIC)

The minimum inhibitory concentration (MIC) is the lowest concentration capable of inhibiting bacterial growth. A solution of 1000 μL was prepared in microtubes (Eppendorf^®^, Hamburg, Germany), consisting of 100 μL of pre-prepared bacterial inoculum and 900 μL of 10% BHI liquid medium. This solution was then distributed into 96-well plates in alphabetical order, with 100 μL in each well. Subsequently, serial microdilutions were performed using 100 μL of MQP oils (WP, SP, WG, and SG). At the end of the microdilution, the oil concentrations ranged from 512 μg/mL to 8 μg/mL. The plates were incubated for 24 h at 37 °C. To determine the MIC, 20 μL of resazurin was added to each well. The redox reaction was observed after one hour, indicated by a color change from blue to red in wells where bacterial growth occurred. The MIC was identified in wells that remained blue, indicating no bacterial growth. The tests were conducted in triplicate [24].

#### 2.2.5. Evaluation of Antibiotic-Enhancing Activity

To evaluate the MQP oils’ ability to enhance antibiotic activity, the method proposed by Coutinho et al. (2008) [25] was used. Following MIC determination with resistant bacteria, subinhibitory concentrations (MIC/8) of the essential oils were determined and combined with antibiotics at concentrations ranging from 512 μg/mL to 0.5 μg/mL. For the tests, 1162 μL of 10% BHI was mixed with 150 μL of the bacterial inoculum and 188 μL of the natural product at a subinhibitory concentration. The control group contained 1350 μL of 10% BHI and 150 μL of bacterial suspension. This solution was distributed into 96-well plates in numerical order (vertical position), with 100 μL in each well. Serial dilutions were then performed using 100 μL of antibiotics, with final concentrations ranging from 512 μg/mL to 0.5 μg/mL. The plates were incubated for 24 h at 37 °C. Subsequently, 20 μL of resazurin was added to measure the antibiotic MIC after combination with the oils. The readings were taken after one hour. The tests were performed in triplicate.

#### 2.2.6. Statistical Analysis

MIC and antibiotic-enhancing activity results were obtained in triplicate, with data expressed as the geometric mean and standard deviation. One-way ANOVA followed by Bonferroni post hoc tests were applied using GraphPad Prism 5.0 software. The results were considered significant at *p* < 0.05.

## 3. Results and Discussion

### 3.1. Relief Characteristics and Climates of the Cities of Paranaguá and Guaratuba

**Figure 2:**
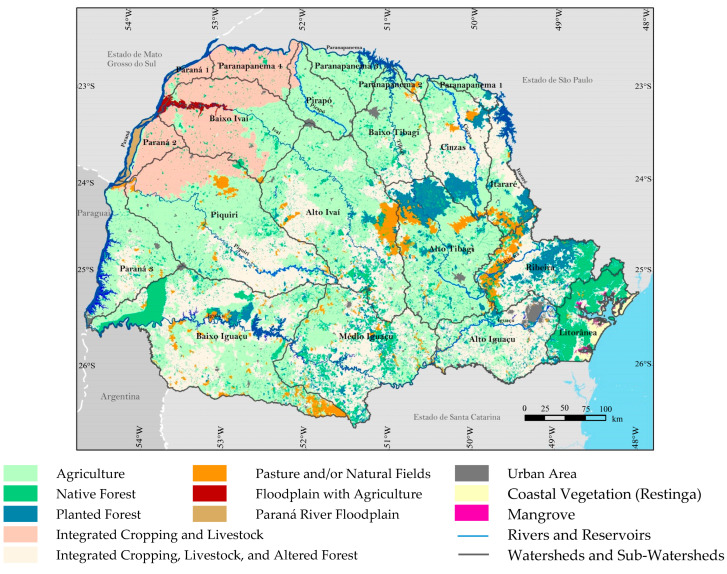
Land use and vegetation cover remnants (2010–2014). Adapted from IPARDES (2015) [29].

**Figure 3:**
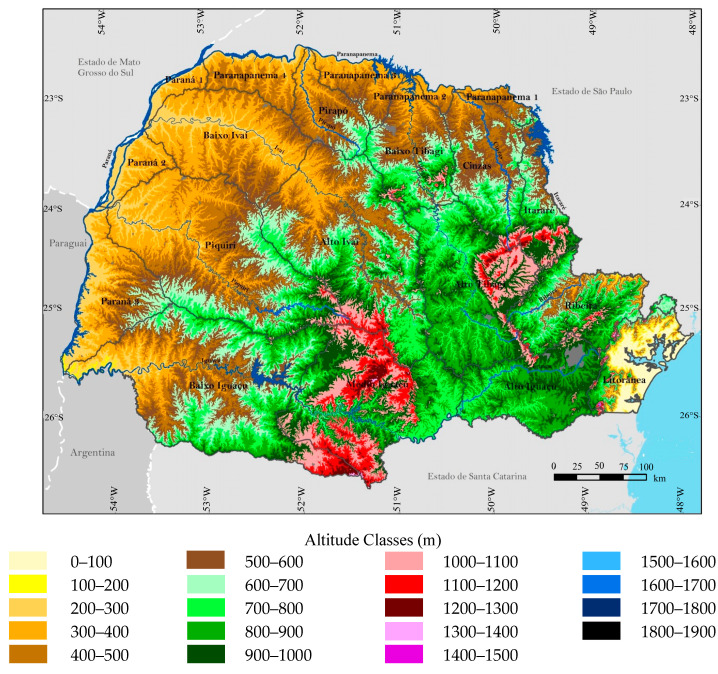
Hypsometry of the State of Paraná. Adapted from IPARDES (2019) [30].

The coastline of the state of Paraná has a geomorphological formation of low altitude that extends to sea level, especially in the cities of Paranaguá and Guaratuba, along the state’s coast. Its relief is mainly characterized by a low-altitude coastal plain; however, higher altitudes can be found in areas such as the Serra do Mar mountain range, with peaks reaching approximately 1877 m (Figure 2 and Figure 3). This relief, with varying altitudes, promotes the existence of microclimates and a diversity of habitats, which are essential for maintaining local biodiversity [26,27,28].

The cities of Paranaguá and Guaratuba experience significant climatic variations, with different precipitation patterns across the seasons. During the summer, which occurs from December to February, there are characteristics of high temperatures, ranging from 20 °C to 31 °C in both cities, along with a significant increase in rainfall, with an average of 226 to 300 mm of precipitation in these cities during the rainiest month, which is January. In the winter, from June to August, temperatures drop, with an average minimum ranging from 11 °C to 13 °C. This variation occurs due to polar air masses, and the climate becomes drier, with a decrease in rainfall, with an average precipitation of 91 mm in August. These climatic changes affect natural irrigation in the winter, making the use of artificial systems necessary in the region [31,32,33].

These geographical and climatic characteristics of the cities are essential for understanding the chemical compositions of propolis and, consequently, of the essential oils, as environmental factors such as topography, temperature, and precipitation directly influence the flora available to bees [34]. Since propolis is formed from plant resins, differences in microclimates between Paranaguá and Guaratuba will impact the bioactive compounds in the collected materials. Therefore, the environmental conditions of these regions provide a fundamental basis for understanding the differences in the chemical composition and biological properties of the analyzed propolis.

### 3.2. Chemical Composition of Essential Oils

Climatic changes significantly influence the composition of bee products due to seasonal variations in the flora available for collection. The coastal region of Paraná presents climatic seasonality between the seasons, which allows variations in the composition of bee products, such as propolis [35,36,37].

The yields of the essential oils varied, with higher values observed in the summer, likely due to the increased availability of productive plant sources and greater bee activity. In Paranaguá, the yield was 1.1% in the summer (SP) compared to 0.4% in the winter (WP). In Guaratuba, a yield of 0.7% was observed in the summer (SG), while in the winter, the value was 0.34% (WG).

The chemical analysis of the essential oils of propolis from *M*. *quadrifasciata quadrifasciata* (OPMQ), conducted by gas chromatography coupled with mass spectrometry (GC-MS), revealed that the characterized volatile compounds represented approximately 93.83% of the WP essential oil, 95.63% of the SP essential oil, 95.56% of the WG essential oil, and 92.04% of the SG essential oil (Table 3). Approximately 68 distinct volatile substances were identified in the analyzed oils.

Analyzing these data, Shehata et al. (2020) [38] and Wagh (2013) [16] explained that these differences in the compositions of different essential oil samples are influenced by factors such as vegetation and climate found in each season, leading to variations in compounds and their respective percentages.

The specific analysis showed that the compounds and quantities of OPMQ primarily varied in two constituents. β-caryophyllene has the highest proportion, 24.55% in the WP oil, while β-bisabolene has 22.87%. Most of the compounds remain the same in the SP oil but in different proportions; the most notable are β-caryophyllene with 18.88% and β-bisabolene with 35.99%. β-bisabolene has 44.56% in the WG oil, while β-caryophyllene is only present in traces, and ar-curcumene has the highest second proportion, at 11.95%. The composition of β-caryophyllene was 21.19%, and β-bisabolene was 25.69% in the SG oil (Table 3).

The major components of the research samples of OPMQ, β-caryophyllene and β-bisabolene, stand out due to their high percentage in the oils. However, it is interesting to note that β-caryophyllene was only found in traces in the WG sample (Table 3). According to some authors, these two major compounds exhibit different pharmacological activities. Notably, we can mention their anti-inflammatory, analgesic, antioxidant, neuroprotective, anticancer, and antimicrobial properties [39,40,41,42,43].

Supporting the findings, previous studies, such as those by Pereira et al. (2020) [44] and Silveira et al. (2022) [45], address the influence of seasonality on the chemical compounds found in essential oils, resulting in various bioactive substances from the same source, collected in different seasons. This differentiation in propolis formation is explained by temperature variation factors and the metabolic activity of plants before biosynthesis [46,47].

According to Sousa et al. (2023) [48], the diversity of compounds found in essential oils is one of the determining factors for their pharmacological activities. This variability can lead to significant differences in the results obtained with each oil, reflecting these differences in biological activities in the formation of each.

### 3.3. Minimum Inhibitory Concentration of Essential Oils

From the antibacterial activity evaluation tests, it was observed that the OPMQ did not show activity at clinically relevant concentrations for the strains *E. coli* ATCC 25,922 and *S*. *aureus* ATCC 25923, and for multidrug-resistant strains of *E. coli* 06 and *S. aureus* 10, where all OPMQ showed MIC ≥ 1024 μg/mL (Table 4.).

Therefore, when tested against clinically relevant and multidrug-resistant bacterial strains, none of the compounds showed significant antibacterial activity. These results suggest that, at concentrations above 1024 μg/mL, the compounds are no longer effective for clinical use due to their low efficacy, as they would require excessively large amounts to be effective [49,50].

### 3.4. Antibiotic Potentiating Activity

**Figure 4:**
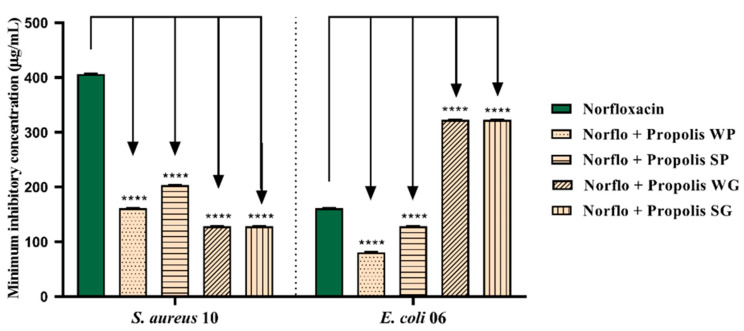
Evaluation of norfloxacin potentiation with OPMQ in *S. aureus* 10 and *E. coli* 06 bacterial strains. Norflo: norfloxacin; WP: Winter Paranaguá; SP: Summer Paranaguá; WG: Winter Guaratuba; SG: Summer Guaratuba. ****: *p* < 0.0001 vs. control antibiotic (statistical difference in relation to the control).

The subinhibitory concentrations of OPMQ in combination with antibiotics were evaluated at concentrations equivalent to MIC/8. In the analysis of the combination of norfloxacin with OPMQ against *S. aureus* 10, a reduction in the MIC of norfloxacin was observed. The MIC decreased from 427 μg/mL to 171 μg/mL when combined with WP; to 213 μg/mL with SP; and to 128 μg/mL with WG and SG. Against the *E. coli* 06 strains, a reduction in the MIC from 171 μg/mL to 85 μg/mL was observed when combined with WP and to 128 μg/mL with SP. However, when combined with oils from WG and SG, an increase in the MIC of norfloxacin was observed, reaching 341 μg/mL (Figure 4).

**Figure 5:**
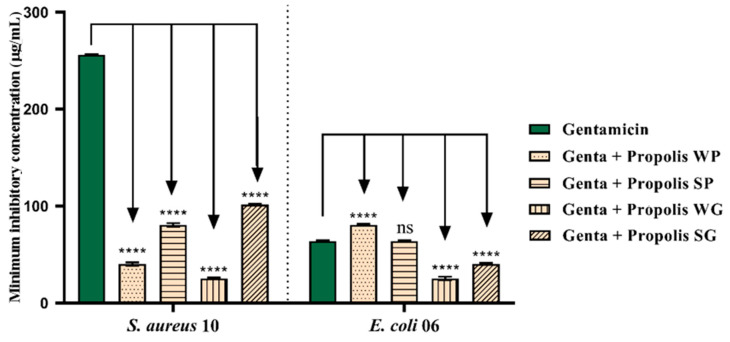
Evaluation of gentamicin potentiation with OPMQ in *S. aureus* 10 and *E. coli* 06 Bacterial Strains. Genta: gentamicin; WP: Winter Paranaguá; SP: Summer Paranaguá; WG: Winter Guaratuba; SG: Summer Guaratuba. ****: *p* < 0.0001 vs. control antibiotic (statistical difference in relation to the control); ns: not significant vs. control antibiotic.

When gentamicin was combined with OPMQ against *S. aureus* 10, a significant reduction in the MIC was observed in all the tested samples. The WP oil reduced the MIC to 59 μg/mL, SP to 117 μg/mL, WG to 26 μg/mL, and SG to 106 μg/mL, representing a reduction of 250 μg/mL compared to the initial MIC. For *E. coli* 06, the combination resulted in a potentiating effect, with a reduction in the antibiotic MIC from 50 μg/mL to 35 μg/mL when combined with WG, and to 43 μg/mL when combined with SG (Figure 5.).

**Figure 6:**
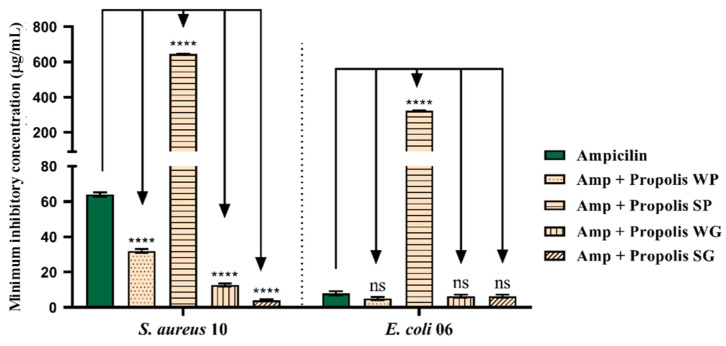
Evaluation of ampicillin potentiation with OPMQ in *S. aureus* 10 and *E. coli* 06 bacterial strains. Amp: ampicillin; WP: Winter Paranaguá; SP: Summer Paranaguá; WG: Winter Guaratuba; SG: Summer Guaratuba. ****: *p* < 0.0001 vs. control antibiotic (statistical difference in relation to the control); ns: not significant vs. control antibiotic.

The association of OPMQ with ampicillin against *S. aureus* 10 showed a significant potentiating effect in WP, WG, and SG, reducing the MIC of ampicillin from 63 μg/mL to 38 μg/mL, 13 μg/mL, and 4 μg/mL, respectively. Against *E. coli* 06, no significant potentiating effect was observed with the tested samples (Figure 6).

Upon conducting a separate and detailed analysis of each essential oil, it was found that the WP oil stood out for potentiating the action of all three antibiotics tested, especially against *S. aureus* 10. The potentiation of the antibiotic activity against *E*. *coli*, particularly with norfloxacin, was also observed. However, WP caused a reduction in antibiotic activity when combined with gentamicin against *E. coli* 06 and showed no significant effect when combined with ampicillin against *E. coli* 06. It is important to analyze its composition, which includes the major components β-caryophyllene (24.55%) and β-bisabolene (22.87%).

Regarding the SP oil, which has β-caryophyllene (18.88%) and β-bisabolene (35.99%) as the major components, it was positively highlighted in its combination with norfloxacin against both bacteria, and in its combination with gentamicin against *S. aureus* 10.

In the analysis of the WG oil against *S. aureus* 10 strains, it potentiated all three classes of antibiotics tested. However, against *E. coli* 06, potentiation was observed only in the combination with gentamicin. The chemical composition of this oil showed β-bisabolene (44.56%) and ar-curcumene (11.95%) as the major components, with β-caryophyllene present only in traces.

Regarding the association of SG oil with antibiotics, similar effects to those observed with WG were noted. The major components of this oil include β-caryophyllene (25.69%) and β-bisabolene (21.19%).

Among the properties of the major compounds, β-caryophyllene has shown antibacterial potential against Gram-positive strains such as *S. aureus*, *Bacillus subtilis*, and *B. cereus*, as well as Gram-negative strains such as *E. coli*, *P. aeruginosa*, and *K. pneumoniae* [38,51,52,53]. Some studies suggest that plant extracts have demonstrated this potential against Gram-negative bacteria when β-caryophyllene is part of the plant’s phytochemical profile [54,55,56], indicating that this compound may exhibit a broader spectrum of action when combined with other phytochemicals.

In the case of β-bisabolene, more studies are needed regarding its antibacterial activity, as it is less commonly discussed in the scientific literature compared to α-bisabolol [57]. However, when present as a major component in certain essential oils, β-bisabolene has shown significant antibacterial activity against *E. coli* [58] and various species of *Streptococcus* and *Mycobacterium* strains [59]. In a study by Li et al. (2023) [42], β-bisabolene exhibited selective antibacterial activity against Gram-positive bacteria in a study involving *Colquhounia coccinea* var. *Mollis*.

Lastly, ar-curcumene, which was present in high concentrations in the WG essential oil, also shows antibacterial potential as discussed in the study by Nascimento et al. (2020) [60]. Studies of essential oils containing ar-curcumene as the major constituent have also demonstrated significant antibacterial activity [61]. However, more research is needed on this compound to better understand its antibacterial properties.

It is evident from this study that the potentiating activity against *S. aureus* 10, a Gram-positive bacterium, showed more significant results in reducing the MIC, unlike the results against *E. coli* 06, a Gram-negative bacterium, where there were results showing reduction, increase, and no significant change in the MIC.

Based on this information, it is important to emphasize the behavior of the OPMQ oils, which showed better performance in potentiating activity against *S. aureus*. These results are supported by observations in the literature, where Bakkali et al. (2008) [11], Nazzaro et al. (2013) [12], Levinson et al. (2018) [62], and Reddy (2019) [63] explain that essential oils face greater resistance to penetration in Gram-negative bacteria due to the presence of the outer layer of lipopolysaccharides [64]. The lipid bilayer and periplasmic space with degradative enzymes present in Gram-negative bacteria also present barriers to antibiotic permeability compared to Gram-positive bacteria [65].

Considering the results of the research, the potentiating activity of OPMQ oils in combination with antibiotics, especially against Gram-positive bacteria, was demonstrated. These findings are in agreement with similar studies using propolis oil from the same bee species [66,67].

Few studies were conducted using essential propolis oil from MQQ. This study is pioneering the evaluation and use of essential oil in the potentiating activity of antibiotics. Other research mainly focuses on the evaluation of MQQ propolis extracts [19,68].

## 4. Conclusions

The study of OPMQ demonstrated that the chemical characterization of the oils revealed different compounds, with similarities in the major compounds, except for one sample (essential oil from the winter of Guaratuba), which contained only traces of a compound (β-caryophyllene) found in the other samples. The analysis of the samples confirmed the influence of seasonality and location on the composition of MQQ propolis. Although propolis is produced by bees, its chemical compounds are influenced by the local vegetation and the season, as demonstrated by the research on samples from the cities of Paranaguá and Guaratuba.

When tested alone, OPMQ did not show significant antibacterial activity but exhibited the ability to potentiate the action of antibiotics, particularly against Gram-positive bacterial strains. The results were modest against Gram-negative strains. The essential oil extracted in the winter of Paranaguá, which showed a chemical composition with the major compounds β-caryophyllene and β-bisabolene, stood out in reducing the MIC of all tested antibiotics, particularly against *S. aureus*, demonstrating a higher capacity to potentiate antibiotics. Therefore, this suggests that OPMQ may be considered adjuvants in antibacterial therapy, improving the effectiveness of antibiotics, especially against Gram-positive bacteria. Further studies are needed to fully understand the potential of these OPMQ and optimize their therapeutic applications.

## Figures and Tables

**Table 1:** Codes for the essential oils of *M*. *quadrifasciata quadrifasciata* (OPMQ).

Sigla	Descrição
OPMQ WP	Oil extracted in Winter from Paranaguá
OPMQ SP	Oil extracted in Summer from Paranaguá
OPMQ WG	Oil extracted in Winter from Guaratuba
OPMQ SG	Oil extracted in Summer from Guaratuba

**Table 2:** Clinical isolates and their resistance profiles.

Bacteria	Origin	Resistance Profile
*Escherichia coli* 06	Urine culture	Cephalexin, Cefoxitin, Cefadroxil, Ceftriaxone, Cefepime, Ampicillin/Sulbactam
*Staphylococcus aureus* 10	Rectal swab culture	Cefadroxil, Cephalexin, Cefalotin, Oxacillin, Penicillin, Ampicillin, Amoxicillin, Moxifloxacin, Ciprofloxacin, Levofloxacin, Ampicillin-Sulbactam, Amoxicillin/Clavulanic Acid, Erythromycin, Clarithromycin, Azithromycin, Clindamycin

Source: Bezerra et al. (2017) [23].

**Table 3:** Main constituents of the essential oils of *M. quadrifasciata quadrifasciata* propolis.

Constituents	RT (min)/FID	RT (min)/EM	RI_exp_/IA_lit_	WP [%]	SP [%]	WG [%]	SG [%]
α-pinene	9330	7125	936/932	0.20	0.93	8.74	1.54
α-copaene	28,088	27,183	1383/1374	7.23	6.28	4.73	5.47
β-caryophyllene	29,999	29,092	1428/1417	24.55	18.88	tr	25.69
α-humulene	31,350	30,475	1462/1452	3.30	3.00	tr	4.74
ar-curcumene	32,383	31,708	1487/1479	4.64	6.91	11.95	4.12
caryophyllene oxide	33,155	32,375	1507/1501	2.19	1.70	-	3.57
β-bisabolene	33,478	32,800	1515/1505	22.87	35.99	44.56	21.19
β-curcumene	33,583	32,933	1518/1514	8.56	7.03	10.95	4.20
δ-cadinene	34,070	33,367	1530/1522	2.85	2.47	1.23	0.70
Others constituents				17.44	12.44	10.40	20.82
Total Characterization				93.83%	95.63%	95.56%	92.04%

WP—Winter Paranaguá; SP—Summer Paranaguá; WG—Winter Guaratuba; SG—Summer Guaratuba; RT (min): retention time in minutes; tr—traces; FID: flame ionization detector. RIexp: Experimental Kovats Index obtained by GC-MS; IAlit: Kovats Index described in the literature.

**Table 4:** Minimum inhibitory concentration of essential oils from *M. quadrifasciata quadrifasciata* propolis.

Bacteria	MIC (μg/mL) of OPMQ			
	WP	SP	WG	SG
* Escherichia coli * ATCC 25922	≥1024	≥1024	≥1024	≥1024
* Staphylococcus aureus * ATCC 25923				
*Escherichia coli 06*				
*Staphylococcus aureus 10*				

WP—Winter Paranaguá; SP—Summer Paranaguá; WG—Winter Guaratuba; SG—Summer Guaratuba.

## Data Availability

Data are contained within the article.

## References

[1] Farrell J.M., Zhao C.Y., Tarquinio K.M., Brown S.P. (2021). Causes and Consequences of COVID-19-Associated Bacterial Infections. Front. Microbiol..

[2] Ikuta K.S., Swetschinski L.R., Robles Aguilar G., Sharara F., Mestrovic T., Gray A.P., Davis Weaver N., Wool E.E., Han C., Gershberg Hayoon A. (2022). Global Mortality Associated with 33 Bacterial Pathogens in 2019: A Systematic Analysis for the Global Burden of Disease Study 2019. Lancet.

[3] O’Neill J. (2016). Tackling Drug-Resistant Infections Globally: Final Report and Recommendations. Review on Antimicrobial Resistance, Wellcome Trust and HM Government. https://amr-review.org/sites/default/files/160525_Final%20paper_with%20cover.pdf.

[4] WHO—World Health Organization World Antimicrobial Awareness Week 2020. https://www.who.int/campaigns/world-antimicrobial-awareness-week/2020.

[5] Murray C.J.L., Ikuta K.S., Sharara F., Swetschinski L., Robles Aguilar G., Gray A., Han C., Bisignano C., Rao P., Wool E. (2022). Global Burden of Bacterial Antimicrobial Resistance in 2019: A Systematic Analysis. Lancet.

[6] WHO, World Health Organization (2020). 2020 Antibacterial Agents in Clinical and Preclinical Development: An Overview and Analysis. https://www.who.int/publications-detail-redirect/9789240021303.

[7] Valli M., Russo H.M., Bolzani V.S. (2018). The Potential Contribution of the Natural Products from Brazilian Biodiversity to Bioeconomy. An. Acad. Bras. Ciênc..

[8] Teotonio R.A. (2018). Farmacognosia.

[9] Sampaio M.A.L., Araújo A.C.J., Borges J.A.O., Lima C.M.G., Coutinho H.D.M., Freitas P.R., Emran T.B., Obaidullah A.J., Silva R.O.M. (2024). Antibacterial and Antibiotic-Modifying Activity of the Commercialized Essential Oil of *Copaifera* spp. Associated with LED Lights Against a *Staphylococcus aureus* Strain. J. Funct. Foods.

[10] Vieira A.J., Beserra F.P., Souza M.C., Totti B.M., Rozza A.L. (2018). Limonene: Aroma of Innovation in Health and Disease. Chem.-Biol. Interact..

[11] Bakkali F., Averbeck S., Averbeck D., Idaomar M. (2008). Biological Effects of Essential Oils—A Review. Food Chem. Toxicol..

[12] Nazzaro F., Frantianni F., de Martino L., Coppola R., de Feo V. (2013). Efeito dos óleos essenciais em bactérias patogênicas. Farmacêuticos.

[13] Mallinger R.E., Gaines-Day H.R., Gratton C. (2017). Do Managed Bees Have Negative Effects on Wild Bees?: A Systematic Review of the Literature. PLoS ONE.

[14] Salatino A., Salatino M.L.F. (2021). Scientific note: Often quoted, but not factual data about propolis composition. Apidologie.

[15] Santos F.D.A.D., Nunes L.E. (2023). Propólis: Aspectos Químicos e Propriedades Terapêuticas. Rev. Eletrônica Multidiscip. Investig. Cient..

[16] Wagh V.D. (2013). Propolis: A Wonder Bees Product and Its Pharmacological Potentials. Adv. Pharmacol. Sci..

[17] Drescher N., Klein A.-M., Schmitt T., Leonhardt S.D. (2019). A Clue on Bee Glue: New Insight into the Sources and Factors Driving Resin Intake in Honeybees (*Apis mellifera*). PLoS ONE.

[18] Lepeletier de Saint Fargeau A.L.M., Brullé A. (1836). Histoire Naturelle des Insectes. Hyménoptères.

[19] dos Santos C.M., Campos J.F., Santos H.F., Balestieri J.B.P., Silva D.B., de Picoli Souza K., dos Santos E.L. (2017). Chemical Composition and Pharmacological Effects of Geopropolis Produced by *Melipona quadrifasciata anthidioides*. Oxidative Med. Cell. Longev..

[20] Torres A.R., Sandjo L.P., Friedemann M.T., Tomazzoli M.M., Maraschin M., Mello C.F., Santos A.R.S. (2018). Chemical Characterization, Antioxidant and Antimicrobial Activity of Propolis Obtained from *Melipona quadrifasciata quadrifasciata* and *Tetragonisca angustula* Stingless Bees. Braz. J. Med. Biol. Res..

[21] Matos F.J.A. (2007). Plantas Medicinais: Guia de Seleção e Emprego de Plantas Usadas na Fitoterapia no Nordeste do Brasil.

[22] Adams R. (2007). Identification of Essential Oils Components by Gas Chromatography/Mass Spectroscopy.

[23] Bezerra C.M., Camilo C.J., Silva M.K.N., Freitas T.S., Ribeiro-Filho J., Coutinho H.D.M. (2017). Vanillin Selectively Modulates the Action of Antibiotics Against Resistant Bacteria. Microb. Pathog..

[24] CLSI—Clinical and Laboratory Standards Institute (2018). Performance Standards for Antimicrobial Susceptibility Testing.

[25] Coutinho H.D., Costa J.G., Lima E.O., Falcão-Silva V.S., Siqueira-Júnior J.P. (2008). Enhancement of the Antibiotic Activity Against a Multiresistant *Escherichia coli* by *Mentha arvensis* L. and Chlorpromazine. Chemotherapy.

[26] Maack R. (1968). Geografia Física do Estado do Paraná.

[27] Silva L.E., Araújo J.P., Amaral W., Menezes E.C.O. (2021). Espécies Nativas Potenciais da Floresta Atlântica do Litoral Paranaense: Gestão de Recursos de Uso Comum no Contexto do Desenvolvimento Territorial Sustentável. Rev. Bras. Meio Ambiente Sustentabilidade.

[28] Silva L.E., Dotto A.R.F., Rebelo R.A. (2022). Bioprospecção e Inovação na Floresta Atlântica: A atuação da REBIFLORA no Litoral do Paraná e Santa Catarina. Rev. Fitos.

[29] IPARDES—Instituto Paranaense de Desenvolvimento Econômico e Social (2015). Uso da Terra Remanescente da Cobertura Vegetal—Paraná (2010–2014). https://www.ipardes.pr.gov.br/sites/ipardes/arquivos_restritos/files/documento/2020-09/Uso%20da%20terra%20e%20Remanescentes%20.pdf.

[30] IPARDES—Instituto Paranaense de Desenvolvimento Econômico e Social (2019). Hipsometria—Paraná. http://www.ipardes.pr.gov.br/sites/ipardes/arquivos_restritos/files/documento/2019-09/Hipsometria%20-%20Paraná.pdf.

[31] Nitsche P.R., Caramori P.H., Ricce W.S., Pinto L.F.D. (2019). Atlas Climático do Estado do Paraná.

[32] Climate Data—Paranaguá Climate (2024). Average Temperature by Month, Paranaguá Water Temperature. https://en.climate-data.org/south-america/brazil/parana/paranagua-3457.

[33] Climate Data—Guaratuba Climate (2024). Average Temperature by Month, Guaratuba Water Temperature. https://en.climate-data.org/south-america/brazil/parana/guaratuba-34671.

[34] Gerginova D., Popova M., Chimshirova R., Trusheva B., Shanahan M., Guzmán M., Solorzano-Gordillo E., López-Roblero E., Spivak M., Simova S. (2023). The Chemical Composition of *Scaptotrigona mexicana* Honey and Propolis Collected in Two Locations: Similarities and Differences. Foods.

[35] Algethami J.S., El-Wahed A.A.A., Elashal M.H., Ahmed H.R., Elshafiey E.H., Omar E.M., Naggar Y.A., Algethami A.F., Shou Q., Alsharif S.M. (2022). Bee Pollen: Clinical Trials and Patent Applications. Nutrients.

[36] Zapata-Hernández G., Gajardo-Rojas M., Calderón-Seguel M., Muñoz A.A., Yáñez K.P., Requier F., Fontúrbel F.E., Ormeño-Arriagada P.I., Arrieta H. (2024). Advances and knowledge gaps on climate change impacts on honey bees and beekeeping: A systematic review. Glob. Change Biol..

[37] de Jongh E.J., Harper S.L., Yamamoto S.S., Wright C.J., Wilkinson C.W., Ghosh S., Otto S.J.G. (2022). One Health, One Hive: A Scoping Review of Honey Bees, Climate Change, Pollutants, and Antimicrobial Resistance. PLoS ONE.

[38] Shehata M.G., Ahmad F.T., Badr A.N., Masry S.H., El-Sohaimy S.A. (2020). Chemical Analysis, Antioxidant, Cytotoxic and Antimicrobial Properties of Propolis from Different Geographic Regions. Ann. Agric. Sci..

[39] Dahham S., Tabana Y., Iqbal M., Ahamed M., Ezzat M., Majid A., Majid A. (2015). The Anticancer, Antioxidant and Antimicrobial Properties of the Sesquiterpene β-Caryophyllene from the Essential Oil of Aquilaria crassna. Molecules.

[40] Shu H.-Z., Peng C., Bu L., Guo L., Liu F., Xiong L. (2021). Bisabolane-type Sesquiterpenoids: Structural Diversity and Biological Activity. Phytochemistry.

[41] Rahimi V.B., Askari V.R. (2022). A Mechanistic Review on Immunomodulatory Effects of Selective Type Two Cannabinoid Receptor Β-caryophyllene. BioFactors.

[42] Li D.S., Shi L.L., Guo K., Luo S.H., Liu Y.C., Chen Y.G., Liu Y., Li S.H. (2023). A New Sesquiterpene Synthase Catalyzing the Formation of (R)-β-Bisabolene from Medicinal Plant *Colquhounia coccinea* var. *mollis* and Its Anti-adipogenic and Antibacterial Activities. Phytochemistry.

[43] Ricardi C., Barachini S., Consoli G., Marazziti D., Polini B., Chiellini G. (2024). Beta-Caryophyllene, a Cannabinoid Receptor Type 2 Selective Agonist, in Emotional and Cognitive Disorders. Int. J. Mol. Sci..

[44] Pereira R.L.S., de Freitas T.S., Freitas P.R., de Araújo A.C.J., Campina F.F., Fidelis K.R., dos Santos H.S. (2020). Seasonality Effects on Antibacterial and Antibiotic Potentiating Activity against Multidrug-Resistant Strains of *Escherichia coli* and *Staphylococcus aureus* and ATR-FTIR Spectra of Essential Oils from *Vitex gardneriana* Leaves. Curr. Microbiol..

[45] Silveira R.M., Carvalho A.F., Bünger M.D.O., Francisca M.D.O., da Costa I.R. (2022). Meta-Analysis of the Influence of Seasonality on the Chemical Composition of Essential Oils from *Myrtaceae* Species. South Afr. J. Bot..

[46] de Souza E.A., Inoue H.T., Fernandes Júnior A., Veiga N., Orsi R.D.O. (2014). Influence of Seasonality and Production Method on the Antibacterial Activity of Propolis. Acta Sci. Anim. Sci..

[47] Calegari M.A., Prasniewski A., Silva C.D., Sado R.Y., Maia F.M.C., Tonial L.M.S., Oldoni T.L.C. (2017). Propolis from Southwest of Paraná Produced by Selected Bees: Influence of Seasonality and Food Supplementation on Antioxidant Activity and Phenolic Profile. An. Acad. Bras. Ciênc..

[48] de Sousa D.P., Damasceno R.O.S., Amorati R., Elshabrawy H.A., de Castro R.D., Bezerra D.P., Nunes V.R.V., Gomes R.C., Lima T.C. (2023). Essential Oils: Chemistry and Pharmacological Activities. Biomolecules.

[49] Martins O.A., Ripoll M.K., Waller S.B., Osório L.G., Gomes A.R., Faria R.O., Meireles M.C.A., de Mello J.R.B. (2021). Métodos de Avaliação Antimicrobiana de Extratos de Diferentes Variedades de *Olea europaea* L.: Revisão de Literatura. Sci. Anim. Health.

[50] Yasir M., Nawaz A., Ghazanfar S., Okla M.K., Chaudhary A., Al W.H., Ajmal M.N., AbdElgawad H., Ahmad Z., Abbas F. (2024). Anti-bacterial Activity of Essential Oils Against Multidrug-Resistant Foodborne Pathogens Isolated from Raw Milk. Braz. J. Biol..

[51] Yoo H.-J., Jwa S.-K. (2018). Inhibitory Effects of β-Caryophyllene on *Streptococcus mutans* Biofilm. Arch. Oral Biol..

[52] Francomano F., Caruso A., Barbarossa A., Fazio A., La Torre C., Ceramella J., Mallamaci R., Saturnino C., Iacopetta D., Sinicropi M.S. (2019). β-Caryophyllene: A Sesquiterpene with Countless Biological Properties. Appl. Sci..

[53] Anusha E., Manogem E.M. (2024). Extraction and Characterization of Bioactive Compounds from Plant, *Michelia champaca* (L.) in Polar Solvents: GC-MS and Antibacterial Properties. Ecol. Environ. Conserv..

[54] Moradlou O., Rabiei Z., Delavari N. (2019). Antibacterial Effects of Carbon Quantum Dots@Hematite Nanostructures Deposited on Titanium Against Gram-Positive and Gram-Negative Bacteria. J. Photochem. Photobiol. A Chem..

[55] Sahu M.C., Dubey D., Rath S., Panda T., Padhy R.N. (2015). Monograph: In Vitro Efficacy of 30 Ethnomedicinal Plants Used by Indian Aborigines Against 6 Multidrug Resistant Gram-Positive Pathogenic Bacteria. Asian Pac. J. Trop. Dis..

[56] Verma R., Pavithra P., Janani V., Charumathi K., Indumathy R., Potala S. (2010). Antibacterial Activity of Plants Used in Indian Herbal Medicine. Int. J. Green Pharm..

[57] Firat Z., Demirci F., Demirci B., Kırmızıbekmez H., Baser K.H.C. (2021). Microbial Transformation of (–)-α-Bisabolol Towards Bioactive Metabolites. Rec. Nat. Prod..

[58] Kazemi M., Rostami H. (2014). Composição Química, Atividades Antimicrobiana e Antioxidante do Óleo Essencial de *Psammogeton canescens*. Nat. Prod. Res..

[59] Fernandes C.C., Magalhães L.G., Martins C.H.G., Santiago M.B., Crotti A.E.M., Andrade P.M.D., Santos T.C.L.D., Miranda M.L.D. (2020). Avaliação In Vitro das Atividades Anticárie, Antimicobacteriana, Antileishmania e Citotóxica dos Óleos Essenciais de *Eremanthus erythropappus* e do α-Bisabolol, Seu Principal Sesquiterpeno. Aust. J. Crop Sci..

[60] do Nascimento P.R.S., da Silva Júnior E.L., Branco A.C.d.S.C. (2020). Aplicações farmacológicas da *Cúrcuma longa* L. como planta medicinal: Uma revisão. Res. Soc. Dev..

[61] Al-Dhahli A.S., Al-Hassani F.A., Alarjani K.M., Yehia H.M., Al Lawati W.M., Hejaz Azmi S.N., Khan S.A. (2020). Essential oil from the rhizomes of the Saudi and Chinese *Zingiber officinale* cultivars: Comparison of chemical composition, antibacterial and molecular docking studies. J. King Saud Univ.-Sci..

[62] Levinson W., Chin-Hong P., Joyce E.A., Nussbaum J., Schwartz B. (2018). Review of Medical Microbiology & Immunology: A Guide to Clinical Infectious Diseases.

[63] Reddy D.N. (2019). Essential Oils Extracted from Medicinal Plants and Their Applications. Natural Bio-Active Compounds.

[64] Almeida R.S., Freitas P.R., Araújo A.C.J., Menezes I.R.A., Santos E.L., Tintino S.R., Moura T.F., Filho J.R., Ferreira V.A., Silva A.C.A. (2020). GC-MS Profile and Enhancement of Antibiotic Activity by the Essential Oil of *Ocotea odorífera* and Safrole: Inhibition of *Staphylococcus aureus* Efflux Pumps. Antibiotics.

[65] do Prado G.S.B., da Costa C.F., Carneiro E.N.A., Silva A.J.A., Siqueira T.S., Caetano L.P.C., de Oliveira Vago L. (2023). Mecanismos de resistência a antibióticos em bactérias Gram-negativas: Novas abordagens terapêuticas. Rev. Ibero-Am. Humanidades Ciênc. Educ..

[66] Valcanaia C.P. (2020). Estudo Sobre o Óleo Volátil de Própolis de Abelhas Nativas sem Ferrão *Melipona q. quadrifasciata* e *Tetragonisca angustula* e Avaliação do seu Potencial Biológico. Master’s Thesis.

[67] Valcanaia C.P., Masote J.B.B., Sommer H.F., Schiquet S., Padilha B., Krepsky L., Paganelli C.J., Borges P.P., Danielli L.J., Apel M.A. (2022). Antimicrobial Activity of Volatile Oils from Brazilian Stingless Bees *Melipona quadrifasciata quadrifasciata* and *Tetragonisca angustula* Propolis. Chem. Biodivers..

[68] Piccinini A., de Sousa M.H.O., dos Santos Diniz Freitas M., Cesca K., de Moura N.F. (2022). Composição química e atividade biológica da própolis de *Melipona quadrifasciata*. Res. Soc. Dev..

